# A Plan for a National Dental Association Divided into Four Co-ordinate District Branches

**Published:** 1896-12

**Authors:** A. H. Thompson

**Affiliations:** Topeka, Kansas.


ARTICLE V.

### A PLAN FOR A NATIONAL DENTAL ASSOCIATION DIVIDED INTO FOUR CO-ORDINATE DISTRICT BRANCHES.

BY A. H. THOMPSON, D. D. S., TOPEKA, KANSAS.

The expediency of uniting the various general dental organizations of the United States into one organic whole has been frequently proposed and discussed, but nothing so far has been accomplished as the outcome of these discussions, While the profession feels the need of one representative general organization which shall be truly national and comprehensive, embracing the whole territory of our great domain, no plan has yet been proposed which seemed to fulfil the requirements, Such an organization must embrace the whole Union, it must be representative, and it must be brought within the reach of all members of the profession.

It has been conclusively demonstrated that it is impossible to convene in one great gathering at any one point at one time, and have a fair representative attendance. The attempt has always been unsuccessful, and must forever remain so, on account of the magnificent distances in our great country and the time, fatigue, and expense involved in long trips. This will always prevent a large representative attendance from all sections in any one single meeting. This is observed in the meetings of the American Dental Association, in which the largest representation is always local.

Therefore the plan herewith proposed is designed with a view of obviating this one great difficulty, and yet to bring large gatherings within reach of all sections, that all he profession of all parts of our land may have the advantages of large gatherings and the valuable material they attract.

The plan is to have district organizations which shall be federated under one central organization, but which shall each meet within the limits of their respective districts at times so arranged that they shall not conflict with one another. By being confined within limited regions they can be readily reached by the profession of that region, and thereby insure larger attendance. Each branch will be representative of its own district—delegates being elected to it from the various State and local societies, as in the American Dental Association at present. Membership in one would be membership in all the other branches, so that a member could attend the meetings of any or all branches and equally at home, like the members of a secret order, the district branches being comparable to the separate lodge organizations.

The district organizations would be [illegible] under a central organization and be co-ordinate parts of one organic whole. Each one will be in touch with every other one and with the whole profession through the central organization. This would consist of a president and a council, say of two members from each district branch. This council would be supreme as regards matters of government, but would not have power to legislate on questions of general importance without the consent of the branches. However, the division of power could be arranged between them as might be for the best interests of harmony and simplicity. That, as well as other matters of detail, could be adjusted without difficulty.

The president of the central organization (or alliance, or association, or federation, as the name might be chosen) would be president of the whole organization. He should be a man of age in practice, who had served his profession well; of distinguished abilities, accomplishments, and achievements, who would be elevated to the position as a distinguishing mark of honor by the profession "would delight to honor."

The presidents of the branches would be vice presidents of the whole organization, and with the other necessary officers would be elected by the branches. They would be entitled to preside at any branch meeting, when present and invited to do so by the regular president of that branch. The general president, however, would have precedence at any branch meeting.

The whole United States can best be divided into four districts, the eastern, southern, central and western, each having its representative organization or branch, which would meet within its borders. There would be, first, the eastern (or Atlantic) district; second, the southern (or Gulf) district; third, the central (or Mississippi) district, and fourth, the western (or Pacific) district. It is noticeable that in three of these districts organizations already exist for the convenience of the profession of those districts, which would seem to indorse the feasibility of the plan. In the Atlantic region we have the present American Dental Association; in the Gulf region we have the Southern Association, and in the Pacific region we have the Pacific Coast Congress. These organizations are already complete, and would only need to accept the plan to become integral parts of the general whole. It would only remain for the profession of the Mississippi basin region to organize a branch to make the system complete.

The branch meetings should be held so as not to conflict with one another, at different seasons of the year. Thus, the Eastern branch could meet in summer (as the American Dental Association does now), the Southern in winter, the Central and Western in spring and autumn, as they might mutually arrange. There would thus be a branch meeting in each quarter of the year, and a member of one could attend all of the meetings, in which he would be entitled to all rights and privileges.

This is the plan in general, the details of which could be worked out to accommodate the general convenience, taking care always to have it as simple as possible and not encumber it with too great a load of the machinery of government.

That there is an increasing desire for larger general meetings, distributed so that they shall be within easy reach of the mass of the profession, is demonstrated by the disposition manifested to organize "joint meetings," "interstate meetings," "dental congresses," etc., which shall attract larger numbers and thus make it worth while for men of talent to present their best work. The usefulness of the old-fashioned State societies is passing away, and their decline is due to the decline of the old-fashioned essay and clinic. The rambling essay of the amateur writer of former days is giving way to the highly wrought monograph of the specialist. The small attendance at the State meetings does not offer sufficient inducement for the specialist to present his best efforts, but he reserves them for the larger meetings. Hence, the latter are organized to attract him, and he in turn attracts a larger attendance, so that they react on each other. The State organizations must still be continued, however, for the business of the profession, dental legislation, etc., so that they cannot be dispensed with entirely. But the days of their scientific usefulness are numbered, for they cannot compete with the larger meetings in attracting the attendance and specialists who make those meetings beneficial.—*Cosmos.*

---

ARTICLE VI.

